# Supplementary material for: Chromothripsis during telomere crisis is independent of NHEJ, and consistent with a replicative origin
Source: Genome Res. 2019 May;29(5):737–49. doi: 10.1101/gr.240705.118 (PMC6499312; doi:10.1101/gr.240705.118)
Supplement: Supplemental Material [file supp_gr.240705.118_Supplemental_file_1.zip › contigs/annotated_contigs/DB52/contig.4.DB52_length_302_mean_cov_4.10264900662.docx]

**DB52_length_302_mean_cov_4.10264900662**

```
GAAGCAAACTCTATCCCTACACTGCGATCTTCCAATATTTGT|ATTAATAAAAAAAAAGATCTTAGGCCGGGCGTGGTGGCTCACGCCT
                                           >chr7:74543681-74543770 + E=3e-10 p=2e-02
GTAATCCCAGCACTTTGGTAGGCCAAGGCAGGAGGATCACTTGAG|CCTGGGAGTTT|AAGACCAGCCTGGGCAATAGAGCAAGACCCC
                                                         >chr1:54813050-54813093 - E=2e
ATCTTTT|TTTTT|GAGATGGAGTCTGGCTCTGTCGCCCAGGCTGGAGTGCAGTGGCGCAATTTCGGCTCACGGCAAGCTCCACCTCCT
-11     >chr11:59235696-59235783 + E=6e-29
GGGTTCA|GG|AAAAACAAACAAAAACCCTGAGGGGGAAAAA          >chr19:58976804-58976835 - E=3
```
